# Short-term variation of HIV tropism readouts in the absence of CCR5 antagonists

**DOI:** 10.1186/1758-2652-13-S4-O9

**Published:** 2010-11-08

**Authors:** CJ Brumme, W Dong, D Chan, T Mo, LC Swenson, C Woods, J Demarest, J Heera, H Valdez, PR Harrigan

**Affiliations:** 1 BC Centre for Excellence in HIV/AIDS, Vancouver, Canada; 2 ViiV Healthcare, Research Triangle Park, USA; 3 Pfizer Global R&D, New London, USA; 4 Pfizer Inc., New York, USA

## Background

Spontaneous tropism changes (from R5 to non-R5 or vice-versa) were observed in approximately 10% of patients between screening and study baseline in the maraviroc (MVC) clinical trials. Little is known of the biology of these apparent short-term tropism fluctuations.

## Methods

Population-based and "deep" V3-loop sequencing were performed in 53 MVC recipients in the MERIT, MOTIVATE and A4001029 studies who spontaneously changed tropism readout by the original Trofile assay between screening and baseline (~4-8 weeks) and 72 randomly sampled patients who did not change. Tropism was inferred by "geno2pheno" with previously defined cutoffs: 2% X4 prevalence with a 3.5% false-positive rate (fpr) for "deep" sequencing; 5.75% fpr for population-based sequencing.

## Results

Patients changing Trofile readout from R5 to non-R5 had significantly higher screening non-R5 prevalence by "deep" sequencing than those who remained R5, and this increased slightly by the baseline timepoint (Table 1). Similarly, patients who changed tropism from non-R5 to R5 in the A4001029 trial had a lower percentage of non-R5 viruses at screening and baseline. Although there was no difference in total viral load, absolute CXCR4-using plasma virus load was higher in those who changed tropism at screening (2.7 vs. 0 log_10_ copies/mL, p=0.02 in MERIT; 3.1 vs. 0 log_10_ copies/mL, p<0.0001 in MOTIVATE) and baseline (3.0 vs. 0 log_10_ copies/mL, p=0.04 in MERIT; 3.8 vs. 0 log_10_ copies/mL, p<0.0001 in MOTIVATE). Non-R5 was reported at screening in 26% and 49% of patients who changed phenotype to non-R5 by population and deep-sequencing, respectively.

**Table 1:** 

	# Genotyped		Screening X4% [IQR]			Baseline X4% [IQR]		
	No Change	Changed	No Change	Changed	p	No Change	Changed	p

MERIT	25	13	0 [0-0]	1.9 [0-3.3]	0.01	0 [0-0.1]	7 [0-16.3]	0.04

MOTIVATE	25	32	0 [0-0.1]	2.4 [0.1-21.3]	0.0001	0 [0-0.1]	9.1 [0.4-32.7]	<0.0001

A4001029	22	8	5.0 [0.2-91.5]	1.8 [0.4-30.6]	0.5	4.3 [0-76.6]	0.4 [0-47.5]	0.3

## Conclusions

In most cases, the prevalence of non-CCR5 usage inferred from "deep" sequencing was stable over the short term between screening and baseline. Where apparent phenotypic tropism changes from R5 to non-R5 occurred, non-R5 virus was generally detectable at the screening timepoint by genotype, coupled with relatively small increases in non-R5 virus by baseline. Small variations in CXCR4-using HIV populations around the phenotypic assay detection limit, rather than coreceptor switch, contributed to apparent tropism switching from R5 to non-R5.

